# Supplementary material for: Barriers and Facilitators to Accessing Mental and Physical Health Care Among Sexual Minority Women: A Qualitative Exploration
Source: Int J Environ Res Public Health. 2025 Jun 19;22(6):965. doi: 10.3390/ijerph22060965 (PMC12193340; doi:10.3390/ijerph22060965)
Supplement: Supplementary file 1 [file ijerph-22-00965-s001.zip › ijerph-3587870-supplementary.pdf]

Supplementary Materials

Interview Outline

Participants:

- At least 25 sexual minority women ages 18-40
- Recruited by contacted past participants who agreed to be contacted for future research

Logistics:

- Interviews will occur via Zoom and last for approximately 45 minutes
- Interviews will be recorded
- The Principal Investigator and trained research assistants (graduate and undergraduate) will conduct the interviews

Format:

1. Welcome
    a. Introductions
    b. Guidelines
        i. Recording information
        ii. Approximate length of interview (45 mins)
    c. Purpose of the study
    d. Overview of topic areas to be covered
2. Healthcare access
    a. Contextual factors
        i. Can you tell me what the LGBTQ presence is like in your community?
        ii. What do you think your community believes about the importance of LGBTQ healthcare access?
        iii. What do those close to you (e.g., friends, family) believe about the importance of LGBTQ healthcare access?
        iv. What are the LGBTQ laws and policies where you live? How do these impact you?
        v. What are the LGBTQ specific health programs in your area? (e.g., outreach, education, community centers)
    b. Individual factors
        i. What are your beliefs about health and healthcare access?
        ii. How do you think your income level has affected your ability to access healthcare?
        iii. Can you tell me about your health insurance history and how that has impacted your healthcare experience?
        iv. Do you have a regular source of care and ability to access that care (e.g., transportation, finances, time off work)?
        v. How do you feel about your overall health?
3. Barriers to seeking and accessing healthcare
    a. Challenges and difficulties in access

i. What are the challenges and difficulties that you face when trying to access physical or mental healthcare?

b. Specific areas of health

i. What areas of health have you had the most difficulty receiving quality treatment for?

c. Negative experiences with healthcare providers

i. Please tell me about any bad or negative experiences you have had with healthcare providers.

d. Discrimination and prejudice in healthcare

i. Please tell me about any times when you have faced discrimination from a healthcare system or individual healthcare provider.

e. Disclosure of sexual identity to providers

i. What have your experiences been like disclosing or not disclosing your sexual identity to healthcare providers?

f. Perceptions of healthcare provider's knowledge of LGBTQ+ concerns

i. How do you feel about the knowledge your healthcare providers do or do not have related to LGBTQ+ health concerns?

4. Facilitators to seeking and accessing healthcare

a. Comfort in seeking out care

i. What kinds of things have made you feel comfortable seeking out physical or mental healthcare?

b. General facilitators

i. What kinds of things have helped you access physical or mental healthcare?

c. Provider(s) facilitators

i. What are some things your provider(s) has done that have been helpful?

ii. What health-related topics do you feel comfortable talking to your provider(s) about?

iii. What are some things your provider(s) could do differently?

d. Individual facilitators

i. Why is it important to you to seek out physical and mental healthcare for yourself?

ii. If you have had negative experiences in sought out care afterwards, what motived you to re-engage in services?

5. Conclusion

a. Provide health-related resources

b. Ask participant if she would like to be contacted with a summary of the results in the future

c. Information about compensation

d. Thank you

Demographic Questionnaire

1. How old are you?

2. Which categories best describe you? If more than one category describes you, select all that apply.
   - ☐ White or Caucasian
     For example: German, Irish, English, Italian, Polish, French, etc.
   - ☐ Black or African American
     For example: African American, Jamaica, Haitian, Nigerian, Ethiopian, Somalian, etc.
   - ☐ American Indian or Alaska Native
     For example: Navajo Nation, Blackfeet Tribe, Mayan, Aztec, Nome Eskimo Community, etc.
   - ☐ Asian or Asian American
     For example: Chinese or Chinese American, Filipino, Asian Indian, Vietnamese, Korean, Japanese, etc.
   - ☐ Middle Eastern or Northern African
     For example: Lebanese, Iranian, Egyptian, Syrian, Moroccan, Algerian, etc.
   - ☐ Native Hawaiian or Other Pacific Islander
     For example: Native Hawaiian, Samoan, Chamorro, Tongan, Fijian, Marshallese, etc.
   - ☐ Other (please specify) ______________________________
   - ☐ Prefer not to answer

3. Do you consider yourself Hispanic, Latinx, or of Spanish origin? (For example, Mexican or Mexican American, Puerto Rican, Cuban, Salvadoran, Dominican, Columbian, Brazilian, etc.)
   - ☐ Yes, I consider myself Hispanic, Latinx, or of Spanish origin
   - ☐ No, I <u>do not</u> consider myself Hispanic, Latinx, or of Spanish origin

4. How would you describe yourself? (select all that apply)
   - ☐ Woman
   - ☐ Man
   - ☐ Trans woman
   - ☐ Trans man
   - ☐ Gender queer/non-conforming
   - ☐ Nonbinary
   - ☐ Other (please specify) ______________________________
   - ☐ Prefer not to answer

5. Which sex were you assigned at birth? (that is, what appears on your birth certificate?)

- ☐ Female
- ☐ Male
- ☐ I don't know
- ☐ Other (please specify) ____
- ☐ Prefer not to answer

6. What best describes your educational level?

- ☐ Less than high school
- ☐ Some high school
- ☐ High school graduate
- ☐ Some college
- ☐ Associate's degree
- ☐ Bachelor's degree
- ☐ Master's degree
- ☐ Doctoral/Professional degree

7. There are many ways that individuals think of their sexual identity. Choose all that describe you:

- ☐ Heterosexual or straight
- ☐ Lesbian
- ☐ Bisexual
- ☐ Queer
- ☐ Asexual
- ☐ Pansexual
- ☐ Questioning
- ☐ Gay
- ☐ Other (specify): ____________________

8. What is your employment status? (check all that apply)

- ☐ Employed part-time
- ☐ Employed full-time (or more)
- ☐ Retired
- ☐ Student
- ☐ Homemaker
- ☐ Unemployed

9. What is your average individual income?

- ☐ $0 - $9,999
- ☐ $10,000 - $19,999
- ☐ $20,000 - $29,999
- ☐ $30,000 - $39,999

- ☐ $40,000 - $49,999
- ☐ $50,000 - $59,999
- ☐ $60,000 - $69,999
- ☐ $70,000 - $79,999
- ☐ $80,000 - $89,999
- ☐ $90,000 - $99,999
- ☐ $100,000+

10. How much are finances an issue for you or your immediate family?

- ☐ Difficulty meeting my/my family's basic needs
- ☐ Barely able to meet my /my family's basic needs
- ☐ Once-in-a-while have difficulty covering my/my family's basic needs
- ☐ No difficulty covering basic needs
- ☐ Have extra money each month

11. How often do you think about your sexual orientation/identity?

- ☐ 1 - Never
- ☐ 2
- ☐ 3
- ☐ 4
- ☐ 5
- ☐ 6 - Often

12. At what age did you first wonder about your sexual identity? ______________________years

13. At what age did you self-identify as being lesbian/gay/bisexual/other? ________________________years

14. At what age did you first disclose your sexual identity to someone else? _________________________years

15. Have you disclosed your sexual identity to a parent or guardian? Yes/No

    If yes: At what age did you first disclose your sexual identity to a parent or guardian?

    ____________years

16. Have you disclosed your sexual identity to another family member other than a parent or guardian? Yes/No

    If "yes": At what age did you first disclose your sexual identity to another family member other than a parent or guardian? ____________________________________years

17. Have you “come out” to any of your friends? Yes/No

If “yes”: At what age did you first "come out" to friends? ______________________years

18. Have you “come out” to any of your coworkers? Yes/No

If “yes” to question 20, then: At what age did you first "come out" to coworkers?

____________years

19. Relative to other lesbian/gay/bisexual individuals, I am:

- ☐ Definitely in the closet.
- ☐ In the closet most of the time.
- ☐ Half-in and half-out.
- ☐ Out of the closet most of the time.
- ☐ Completely out of the closet.
- ☐ Prefer not to answer

20. **During the past year**, with whom have you had sex?

- ☐ Woman/women
- ☐ Man/men
- ☐ Other
- ☐ No one
- ☐ Prefer not to answer

21. With whom have you had sex in your **lifetime**?

- ☐ Woman/women
- ☐ Man/men
- ☐ Other
- ☐ No one
- ☐ Prefer not to answer

22. How would you describe your relationship status?

- ☐ Single (not dating anyone)
- ☐ Dating one partner
- ☐ Dating several partners
- ☐ In a monogamous relationship

- ☐ In an open relationship
- ☐ Polyamorous
- ☐ Engaged, married, or in a civil union
- ☐ Other: ____________________________

<u>If not single</u>: Is the other person (or people) you are dating or in a relationship with: (check all that apply)

(1) A woman (or women)
(2) A man (or men)
(3) A gender non-binary/genderqueer individual(s)
(4) Other: __________________

<u>If not single</u>: How long have you been in your current relationship? ___________________Years

_______Months

23. What is your height? ________________ Feet _________________Inches

24. What is your best guess of your <u>current weight</u> in pounds? ______________________

What is your best guess of your <u>highest adult weight</u> in pounds? ___
